# Deciphering a pathway of *Halobacterium salinarum* N-glycosylation

**DOI:** 10.1002/mbo3.215

**Published:** 2014-12-02

**Authors:** Lina Kandiba, Jerry Eichler

**Affiliations:** Department of Life Sciences, Ben Gurion University of the NegevBeersheva, Israel

**Keywords:** Archaea, halophile, N-glycosylation, posttranslational modification

## Abstract

Genomic analysis points to N-glycosylation as being a common posttranslational modification in Archaea. To date, however, pathways of archaeal N-glycosylation have only been described for few species. With this in mind, the similarities of N-linked glycans decorating glycoproteins in the haloarchaea *Haloferax volcanii* and *Halobacterium salinarum* directed a series of bioinformatics, genetic, and biochemical experiments designed to describe that *Hbt. salinarum* pathway responsible for biogenesis of one of the two N-linked oligosaccharides described in this species. As in *Hfx. volcanii*, where *agl* (*a*rchaeal *gl*ycosylation) genes that encode proteins responsible for the assembly and attachment of a pentasaccharide to target protein Asn residues are clustered in the genome, *Hbt. salinarum* also contains a group of clustered homologous genes (*VNG1048G*-*VNG1068G*). Introduction of these *Hbt. salinarum* genes into *Hfx. volcanii* mutant strains deleted of the homologous sequence restored the lost activity. Moreover, transcription of the *Hbt. salinarum* genes in the native host, as well as in vitro biochemical confirmation of the predicted functions of several of the products of these genes provided further support for assignments made following bioinformatics and genetic experiments. Based on the results obtained in this study, the first description of an N-glycosylation pathway in *Hbt. salinarum* is offered.

## Introduction

Once thought restricted to Eukarya, it is now clear that N-glycosylation is a posttranslational modification performed across evolution (Larkin and Imperiali 2011; Aebi 2013; Eichler 2013; Nothaft and Szymanski 2013; Jarrell et al. 2014). In each of the three domains, N-glycosylation involves the assembly of lipid-linked oligosaccharides that are subsequently transferred to target Asn resides. However, it is the archaeal version of this universal protein-processing event that generates an unparalleled degree of diversity, relative to what is seen in Eukarya or Bacteria. This variety is apparent in the composition, size, and degrees of saturation and phosphorylation of the lipid carrier upon which glycans are assembled, in terms of glycan architecture and sugar content, and with respect to the identity of the linking sugar that connects the glycan to the lipid carrier or the target Asn residue (Eichler 2013; Jarrell et al. 2014). Indeed, given that N-glycosylation is seemingly a common event in Archaea (Kaminski et al. 2013), the diversity observed in the limited number of archaeal N-glycosylation pathways and N-linked glycans that have been characterized to date (Eichler 2013) likely represents the tip of an iceberg. To better understand the origins of such diversity, it will be necessary to delineate pathways of N-glycosylation across the Archaea. Largely due to the technical challenges associated with growing the majority of known archaeal strains in the laboratory, as well as the relatively few number of strains for which molecular tools have been developed, progress in describing pathways of archaeal N-glycosylation has been slow. Today, pathways of N-glycosylation have been defined to varying degrees of detail only in the halophile *Haloferax volcanii*, in the methanogens *Methanococcus maripaludis* and *Methanococcus voltae* and in the thermoacidophile *Sulfolobus acidocaldarius* (for review, see Jarrell et al. 2014).

In *Hfx. volcanii*, glycoproteins are modified by an N-linked pentasaccharide comprising a hexose, two hexuronic acids, a methyl ester of a hexuronic acid and a mannose via a series of Agl (*a*rchaeal *gl*ycosylation) proteins (Abu-Qarn et al. 2007; Guan et al. 2010; Magidovich et al. 2010). Assembly of the pentasaccharide involves the addition of the first four sugars of the pentasaccharide to a common dolichol phosphate carrier on the cytoplasmic face of the plasma membrane by the sequential actions of the glycosyltransferases AglJ, AglG, AglI, and AglE (Abu-Qarn et al. 2008; Plavner and Eichler 2008; Yurist-Doutsch et al. 2008; Kaminski et al. 2010). Other pathway components, including AglF, a glucose-1-phosphate uridyltransferase (Yurist-Doutsch et al. 2010), AglM, a UDP-glucose dehydrogenase (Yurist-Doutsch et al. 2010), AglP, a methyltransferase (Magidovich et al. 2010) and AglQ, an apparent epimerase (Arbiv et al. 2013), also contribute to the assembly of the dolichol phosphate-linked tetrasaccharide. In parallel, the final pentasaccharide subunit, mannose, is added to its own dolichol phosphate carrier by the glycosyltransferase AglD (Abu-Qarn et al. 2007; Guan et al. 2010). Once assembled, the lipid-charged glycans are translocated across the membrane by an unknown mechanism, although AglR is apparently involved in the process (Kaminski et al. 2012). At this point, the oligosaccharyltransferase AglB (Abu-Qarn and Eichler 2006; Abu-Qarn et al. 2007) delivers the translocated tetrasaccharide and its precursors from the lipid carrier to select Asn residues of the glycoprotein. Finally, the terminal mannose is transferred from its ‘flipped’ lipid carrier to the protein-bound tetrasaccharide by AglS (Cohen-Rosenzweig et al. 2012).

While considerable attention has focused on N-glycosylation in *Hfx. volcanii*, the first example of this posttranslational modification in Archaea and, indeed, beyond the Eukarya, was provided by another haloarchaeon, *Halobacterium salinarum* (Mescher and Strominger 1976). Two *Hbt. salinarum* proteins are known to be N-glycosylated, namely the S-layer glycoprotein and archaellin, with the former being modified by two distinct N-linked glycans (Wieland 1988; Lechner and Wieland 1989). The N-linked glycan common to both the S-layer glycoprotein and archaellin corresponds to a glucose, three glucuronic acids and a glucose, although the presence of a glucose and three glucuronic acids has also been reported (Lechner et al. 1985a,b; Wieland et al. 1985; Wieland 1988). As such, the structure of this *Hbt. salinarum* N-linked glycan is reminiscent of its *Hfx. volcanii* counterpart. At the same time, the glucuronic acids of the *Hbt. salinarum* N-linked glycan, a third of which are replaced by the isomer iduronic acid, are sulfated (Lechner et al. 1985a; Wieland et al. 1986).

Presently, only little is known of the process of N-glycosylation in *Hbt. salinarum*. As in *Hfx. volcanii*, the N-linked pentasaccharide of *Hbt. salinarum* is assembled on a dolichol phosphate carrier, at which stage sulfation also takes place (Lechner et al. 1985a). However, in contrast to what occurs in *Hfx. volcanii*, where both the lipid- and the protein-linked glycans are methylated (Magidovich et al. 2010), the *Hbt. salinarum* glycan presents a methyl group at the nonreducing end glucose only when bound to dolichol phosphate and not when attached to the target protein, suggesting that in *Hbt. salinarum*, such transient methylation is important for delivery of the lipid-linked glycan across the membrane (Lechner et al. 1985b). Moreover, constituent iduronic acids are already detected at the lipid-linked glycan stage and not only at the protein-bound stage, as is the case in eukaryotes (Wieland et al. 1986). Finally, the actual N-glycosylation event in *Hbt. salinarum* was shown to occur on the outer surface of the cell (Lechner et al. 1985b).

As in *Hfx. volcanii*, the *Hbt. salinarum* genome contains a single *aglB* gene encoding the archaeal oligosaccharyltransferase (Magidovich and Eichler 2009; Kaminski et al. 2013). Examination of the genes adjacent to *Hbt. salinarum aglB* reveals the presence of a cluster of sequences annotated as serving glycosylation-related roles, often homologous to *Hfx. volcanii agl* genes (Yurist-Doutsch and Eichler 2009; Kaminski et al. 2013). By exploiting the predicted similarities between *Hfx. volcanii* Agl proteins and their *Hbt. salinarum* counterparts and then confirming those predictions experimentally, the present study provides the first description of a pathway responsible for N-glycosylation in *Hbt. salinarum*.

## Experimental Procedures

### Cell growth

*Hfx. volcanii* cells deleted of *aglD*, *aglE*, *aglF*, *aglG*, *aglI*, *aglJ*, *aglM*, *aglP*, *aglQ*, or *aglR* were grown in complete medium containing 3.4 mol/L NaCl, 0.15 mol/L MgSO_4_·7H_2_0, 1 mmol/L MnCl_2_, 4 mmol/L KCl, 3 mmol/L CaCl_2_, 0.3% (w/v) yeast extract, 0.5% (w/v) tryptone, 50 mmol/L Tris-HCl, pH 7.2, at 40°C (Abu-Qarn et al. 2007, 2008; Yurist-Doutsch et al. 2008, 2010; Kaminski et al. 2010, 2012; Magidovich et al. 2010; Arbiv et al. 2013).

### Plasmid preparation

To generate plasmids encoding *Clostridium thermocellum* cellulose-binding domain (CBD)-tagged VNG1053G, VNG1065C, VNG1058H, VNG1062G, VNG1054G, VNG1055G, VNG1066C, VNG1067G, VNG1048G, and VNG0318G, these genes were PCR-amplified from the *Hbt. salinarum* (*Halobacterium* sp. NRC-1) genome using primers designed to introduce restriction sites at the start and the end of each sequence (primers and restriction sites are listed in Table S3). The amplified fragments were digested with appropriate restriction enzymes and ligated into plasmid pWL-CBD (Morag et al. 1995), previously digested with the same restriction enzymes. The plasmids were then introduced into *Hfx. volcanii* cells deleted of *aglD*, *aglE*, *aglF*, *aglG*, *aglI*, *aglJ*, *aglM*, *aglP*, *aglQ*, or *aglR* as described in the text.

### Reverse transcriptase-polymerase chain reaction

Reverse transcriptase-polymerase chain reaction (RT-PCR) was performed as described previously (Abu-Qarn and Eichler 2006). Briefly, specific forward and reverse oligonucleotide primers were designed for each *Hbt. salinarum* gene under consideration (Table S3). RNA isolation was carried out using TRIzol reagent (Invitrogen, Carlsbad, CA). RNA concentration was determined spectrophotometrically. After contaminating DNA was eliminated with a DNA-Free kit (Ambion, Austin, TX), single-stranded cDNA was prepared for each sequence from the corresponding RNA (2 *μ*g) using a High Capacity cDNA Reverse Transcription kit (Applied Biosystems, Foster City, CA). The cDNA was then used for PCR amplification, together with appropriate forward and reverse primer pairs. cDNA amplification was monitored by electrophoresis in 1% agarose gels. The sequences of the PCR products were determined to confirm their identity. In control experiments designed to exclude any contribution from contaminating DNA, PCR amplification was performed on total RNA prior to cDNA preparation.

### Liquid chromatography-electrospray ionization mass spectrometry (LC-ESI MS)

Liquid chromatography-electrospray ionization mass spectrometry (LC-ESI MS) analysis of the *Hfx. volcanii* S-layer glycoprotein was performed as described (Kaminski et al. 2010).

### Protein purification

CBD-tagged proteins were purified as previously described (Irihimovitch et al. 2003). Briefly, 1 mL aliquots of *Hfx. volcanii* cells transformed to express CBD-VNG1048G or CBD-VNG1055G were grown to mid-logarithmic phase, harvested, and resuspended in 1 mL solubilization buffer (1% Triton X-100, 3.5 mol/L NaCl, 50 mmol/L Tris-HCl, pH 7.2) containing 3 *μ*g/mL DNaseI and 0.5 *μ*g/mL PMSF. The solubilized mixture was nutated for 20 min at 4°C, after which time 50 *μ*L of a 10% (w/v) solution of cellulose was added. After a 120 min nutation at 4°C, the suspension was centrifuged (2655 ***g*** for 5 min), the supernatant was discarded and the cellulose pellet was washed four times with wash buffer containing 3.5 mol/L NaCl, 50 mmol/L Tris-HCl, pH 7.2. After the final wash, the cellulose beads were centrifuged (2655 ***g*** for 5 min), the supernatant was removed and the pellet, containing cellulose beads linked to the CBD-tagged proteins, was employed in various in vitro assays.

### UDP-glucose dehydrogenase activity assay

The UDP-glucose dehydrogenase activity of CBD-VNG1048G was assayed in reaction buffer (3.5 mol/L NaCl, 5 mmol/L MgCl_2_, 50 mmol/L Tris-HCl, pH 9.0), essentially as described previously (Yurist-Doutsch et al. 2010).

### Glucose-1-phosphate nucleotidyltransferase activity assay

To test for glucose-1-phosphate nucleotidyltransferase activity, cellulose-bound CBD-VNG1055G was resuspended in reaction buffer was incubated with 5 mmol/L glucose-1-phosphate and 5 mmol/L UTP or dTTP. Aliquots were removed immediately following substrate addition and following incubation at 42°C. After a 10 min at room temperature with 1 U/*μ*L of pyrophosphatase, the extent of phosphate release was determined using a malachite green-based assay (Lanzetta et al. 1979).

## Results

### Deleted *Hfx. volcanii agl* genes can be functionally replaced by their *Hbt. salinarum* homologues

**Figure 1:**
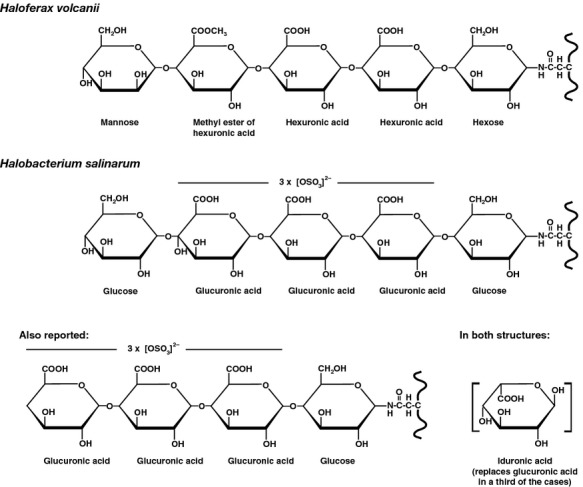
Schematic depiction of N-linked glycans attached to glycoproteins in *Haloferax volcanii* and *Halobacterium salinarum*. In the *Hbt. salinarum* glycan, a third of the glucuronic acids are replaced by iduronic acids (in brackets). See text for details.

**Figure 2:**
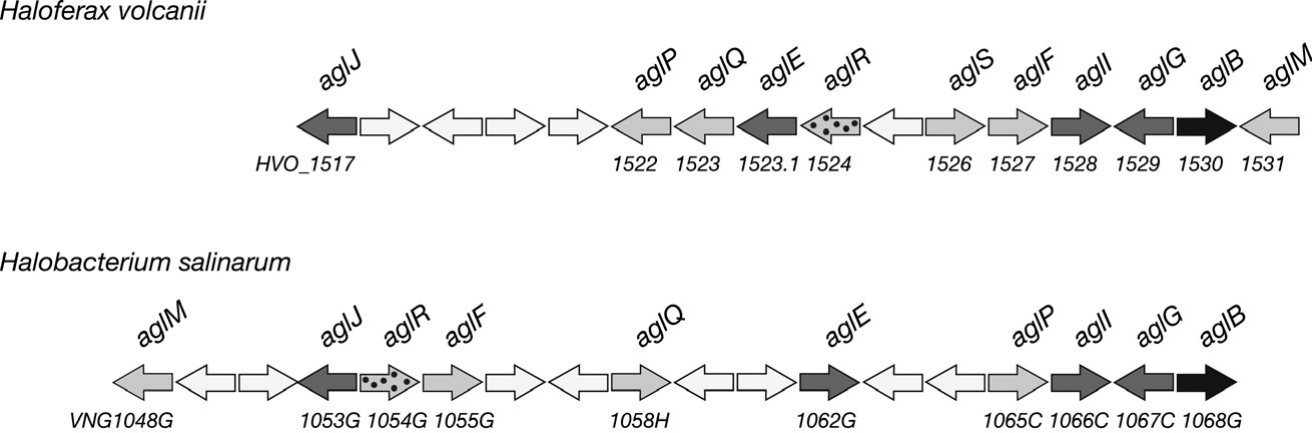
Schematic depiction of the *Haloferax volcanii agl* gene cluster and the comparable region of the *Halobacterium salinarum* genome. Genes encoding glycosyltransferases are in dark gray, genes encoding proteins with glycosylation-related functions are in light gray, genes encoding flippases or flippase-associated proteins are speckled and genes encoding the oligosaccharyltransferase AglB are in black. Transposases and nonglycosylation-related sequences are in white. Gene lengths are arbitrarily drawn.

In *Hfx. volcanii*, all but one of the *agl* genes involved in the assembly and attachment of the N-linked pentasaccharide decorating glycoproteins in this species are found within an *aglB*-based gene cluster (Yurist-Doutsch and Eichler 2009; Yurist-Doutsch et al. 2010). Similarly, *Hbt. salinarum aglB* also anchors a cluster of genes annotated as serving glycosylation-related roles (Table S1). As a first step in determining which, if any, of the products of these *Hbt. salinarum* sequences serves a similar function as do *Hfx. volcanii* Agl proteins, given the similarity of N-linked glycans in the two species (Fig1), each *Hbt. salinarum* gene in this cluster was used as query in a BLAST search of the *Hfx. volcanii* genome at the deduced amino acid sequence level. Based on the results of such searches (Table S2), the various *Hbt. salinarum* genes considered were deemed to be homologues of *Hfx. volcanii agl* genes (Fig2).

**Figure 3:**
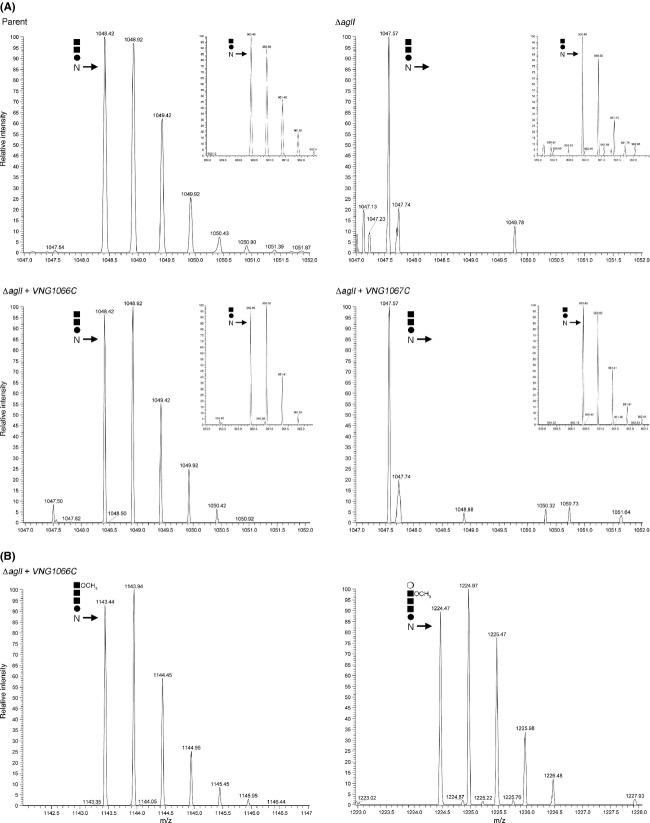
LC-ESI MS reveals the functional replacement of *Haloferax volcanii* AglI by its *Halobacterium salinarum* homologue, VNG1066C. (A) LC-ESI MS profiles showing [M+2H]^2+^ ion peaks corresponding to an Asn-13-containing S-layer glycoprotein tryptic fragment from parent strain cells (upper left panel), from *ΔaglI* cells (upper right panel), from *ΔaglI* cells transformed to express *Hbt. salinarum* VNG1066C (lower left panel) and from *ΔaglI* cells transformed to express *Hbt. salinarum* VNG1067C (lower right panel). In each panel, the position of the trisaccharide-modified peptide (m/z) is indicated, while each inset shows the presence of the disaccharide-modified peptide. (B) *ΔaglI* cells transformed to express *Hbt. salinarum* VNG1066C also modify S-layer glycoprotein Asn-13 with the first four pentsaccharide sugars (left panel) and the complete pentasaccharide (right panel). Hexose is represented by a full circle, hexuronic acid is represented by a full square, and mannose is represented by an open circle.

To confirm these bioinformatics-based predictions, the ability of each *Hbt. salinarum* sequence to replace its *Hfx. volcanii* Agl protein counterpart was considered. To do so, *Hfx. volcanii* cells deleted of a given *agl* gene were transformed to express the corresponding *Hbt. salinarum* homologue bearing a CBD tag. Functional replacement of the deleted gene was assessed based on the ability of the transformed strains to decorate the S-layer glycoprotein with the same N-linked pentasaccharide as detected in the *Hfx. volcanii* parent strain. Accordingly, the S-layer glycoprotein from each transformed strain was treated with trypsin and the LC-ESI MS profile of a peptide containing Asn-13, a position previously shown to be modified by addition of a pentasaccharide and its precursors (Abu-Qarn et al. 2007), was assessed. The results of one such experiment in which *Hfx. volcanii ΔaglI* cells were transformed to express a CBD-tagged version of VNG1066C, the predicted *Hbt. salinarum* homologue of AglI, are presented (Fig3).

Initially, the effect of *Hfx. volcanii aglI* deletion on N-linked pentasaccharide biosynthesis was confirmed. The LC-ESI MS profile obtained from parent strain cells included a [M+2H]^2+^ ion peak at *m/z* 1048.42, corresponding to an S-layer glycoprotein Asn-13-containing fragment modified by the trisaccharide precursor of the N-linked pentasaccharide normally found at this position (Fig3A, upper left panel). In contrast, no such peak was detected in the *ΔaglI* cells (Fig3A, upper right panel), although the disaccharide-modified precursor was detected, as in the parent strain ([M+2H]^2+^ ion peak at *m/z* 960.40; inset in each panel), as previously shown (Yurist-Doutsch et al. 2010). When, however, *Hfx. volcanii Δagl* cells were transformed to express *Hbt. salinarum* VNG1066C, the monoisotopic peak corresponding to the trisaccharide precursor of the Asn-13-linked pentasaccharide was observed (Fig3A, lower left panel). On the other hand, introduction of *Hbt. salinarum* VNG1067C, the predicted homologue of AglG, namely the *Hfx. volcanii* glycosyltransferase responsible for adding the second sugar of the dolichol phosphate-bound tetrasaccharide precursor of the complete N-linked pentasaccharide (Yurist-Doutsch et al. 2008), could not replace the missing activity of the *Hfx. volcanii ΔaglI* cells (Fig3A, lower right panel). In both engineered strains, disaccharide-charged Asn-13-containing peptides were detected (inset of both lower panels). Confirmation that the sugar added to the third position of N-linked pentasaccharide precursor generated in *Hfx. volcanii ΔaglI* cells transformed to express *Hbt. salinarum* VNG1066C corresponds to the same or a similar sugar as added in the parent strain was next sought. LC-ESI MS analysis revealed the presence of [M+2H]^2+^ ion peaks at *m/z* 1143.44 and 1224.47, corresponding to the Asn-13-containing S-layer glycoprotein-derived peptide modified by the first four sugars of the N-linked pentasaccharide and by the complete pentasaccharide, respectively (Fig3B, left and right panels, respectively). As such, it can be concluded that *Hbt. salinarum* VNG1066C can functionally replace *Hfx. volcanii* AglI.

### Specificity of glycosyltransferase replacement

In assigning which *Hbt. salinarum* genes encode homologues of the *Hfx. volcanii* glycosyltransferases AglJ, AglG, AglI, and AglE, that *Hbt. salinarum* sequence identified with the lowest E-value in BLAST searches was selected in each case (Table S2). However, since homology comparison-based bioinformatics tools are only of limited use for defining the precise substrate of a given glycosyltransferase, the ability of each of the predicted *Hbt. salinarum* glycosyltransferases in the cluster spanning *VNG1048G*-*VNG1068G* to functionally replace *Hfx. volcanii* AglJ, AglG, AglI, or AglE was tested. As noted above, *Hbt. salinarum* VNG1066C could replace its *Hfx. volcanii* homologue, AglI, whereas the homologue of AglG, VNG1067, could not. Similarly, VNG1053G and VNG1062G, the homologues of AglJ and AglE, respectively, could not replace AglI (Table1), with only an N-linked disaccharide being detected in such transformed strains. Likewise, while AglG could be replaced by its homologue, VNG1067C, the homologues of AglJ, AglI, or AglE (VNG1053G, VNG1066C, and VNG1062G, respectively) could not restore the absent activity to the deletion strain, with only monosaccharide-charged Asn-13 being detected. Moreover, although AglE could be replaced by its homologue (VNG1062G), the introduction of the *Hbt. salinarum* AglG or AglI homologues (VNG1066C and VNG1067C, respectively) did not lead to the appearance of the N-linked pentasaccharide in *Hfx. volcanii ΔaglE* cells, with only the N-linked trisaccharide being seen. Finally, the ability of *Hbt. salinarum* VNG1068G to replace its *Hfx. volcanii* homologue, AglB, has been previously shown (Cohen-Rosenzweig et al. 2014).

**Table 1:** Functional replacement of *Haloferax volcanii* Agl proteins by their *Halobacterium salinarum* homologues.

*Halobacterium salinarum*	*Haloferax volcanii*										
	*ΔaglJ*	*ΔaglP*	*ΔaglQ*	*ΔaglE*	*ΔaglR*	*ΔaglF*	*ΔaglI*	*ΔaglG*	*ΔaglB*	*ΔaglM*	*ΔaglD*
VNG1048G										+	
VNG1053G	+			+			−	−			
VNG1054G					+						
VNG1055G						+					
VNG1058H			+								
VNG1062G	+			+			−	−			
VNG1065C		+									
VNG1066C	+			−			+	−			
VNG1067C	+			−			−	+			
VNG1068G									+		
VNG0318G											+

Still, some promiscuity in glycosyltransferase function was observed. The complete pentasaccharide was detected on the Asn-13-containing peptide when *Hfx. volcanii* cells deleted of *ΔaglJ*, encoding the glycosyltransferase responsible for adding the first pentasaccharide sugar to the dolichol phosphate carrier (Kaminski et al. 2010), were transformed to express the *Hbt. salinarum* AglJ homologue VNG1053C but also when the same deletion strain cells were transformed to express VNG1062G, VNG1066C, or VNG1067G, corresponding to the homologues of AglE, AglI, and AglG, respectively. Similarly, both *Hbt. salinarum* VNG1053C (AglJ) and VNG1062G (AglE) could functionally replace *Hfx. volcanii* AglE in cells lacking the encoding gene. However, in *ΔaglE* cells transformed to express VNG1053C, only the first four pentasaccharide sugars decorated Asn-13; no complete pentasaccharide was detected.

### Functional replacement of other *Hfx. volcanii* Agl proteins by their *Hbt. salinarum* homologues

Next, other *Hfx. volcanii* strains lacking a given *agl* gene were transformed to express their predicted *Hbt. salinarum* homologue to determine whether here too the missing activity could be functionally replaced. Such experiments revealed that when *Hfx. volcanii* cells in which *aglM*, *aglR*, *aglF*, and *aglQ* were respectively replaced by *VNG1048G*, *VNG1054G*, *VNG1055*, and *VNG1058H*, the complete pentasaccharide attached to S-layer glycoprotein Asn-13 was generated (Table1). Finally, although functional replacement of the absent methyltransferase activity in *Hfx. volcanii* cells lacking AglP was realized upon introduction of VNG1065C (Fig S1A), the most prominent N-linked glycan in this engineered strain was a tetrasaccharide comprising the first three pentasaccharide sugars and a fourth nonmethylated hexuronic acid (Fig S1B and C). No complete N-linked pentasaccharide was detected in this strain. Thus, despite the fact that both are thought to serve the same function, VNG1065C did not fully restore missing AglP activity in *Hfx. volcanii ΔaglP* cells. Still, AglP function in the mutant cells was restored upon introduction of a plasmid-encoded version of *aglP*, although the efficiency of such complementation was not assessed (not shown). The limited ability of VNG1065C to functionally replace AglP methyltransferase activity may be due to the fact that the CBD-tagged version of the *Hbt. salinarum* AglP homologue was poorly expressed in the *ΔaglP* host strain, as revealed by immunoblot analysis using anti-CBD antibodies (Fig S1D) or due to differential activities of the two proteins.

### VNG0318G adds the final pentasaccharide hexose in *Hfx. volcanii ΔaglD* cells

Previous studies on *Hbt. salinarum* reported the sulfated N-linked glycan detected at both the dolichol phosphate and target protein levels to correspond to either a tetrasaccharide or a pentasaccharide (Lechner and Wieland 1989). Since the *Hbt. salinarum* gene cluster containing homologues of *Hfx. volcanii agl* genes (i.e., *VNG1048G*-*VNG1068G*) only encodes four glycosyltransferases (VNG1053C, VNG1062G, VNG1066C, and VNG1067G), the rest of the *Hbt. salinarum* genome was scanned for a homologue of AglD, that glycosyltransferase responsible for adding the fifth pentasaccharide sugar of the *Hfx. volcanii* N-linked glycan (Abu-Qarn et al. 2007). It was hypothesized that this additional *Hbt. salinarum* glycosyltransferase would be responsible for adding the fifth sugar to the N-linked pentasaccharide in this species. Accordingly, a deduced amino acid-based BLAST search of the *Hbt. salinarum* genome using *Hfx. volcanii* AglD (HVO_0798) as query identified VNG0318G (E-value, 0; score, 724; % coverage, 96; identity 63%) as an AglD homologue. Examination of genes upstream and downstream of *Hfx. volcanii aglD* and *Hbt. salinarum VNG0318G* revealed a stretch of *Hfx. volcanii* genes spanning from *HVO_0780*-*HVO_0812* that was essentially mirrored by *Hbt. salinarum* genes spanning from *VNG0298H-VNG0330G* (Fig S2). Indeed, of the 22 homologous gene pairs in these stretches, the predicted protein products share identities at levels ranging from 39% to 82%. To determine whether VNG0318G could functionally replace AglD, *Hfx. volcanii ΔaglD* cells were transformed to express CBD-tagged VNG0318G. A tryptic fragment of the S-layer glycoprotein containing Asn-13 was then examined by LC-ESI MS. Whereas only peptide modified by the first four pentasaccharide sugars was detected in the deletion strain, the same cells transformed to express VNG0318G added a complete pentasaccharide to S-layer glycoprotein Asn-13 (Fig S3).

### *Hbt. salinarum agl* gene homologues are transcribed in the native host

**Figure 4:**
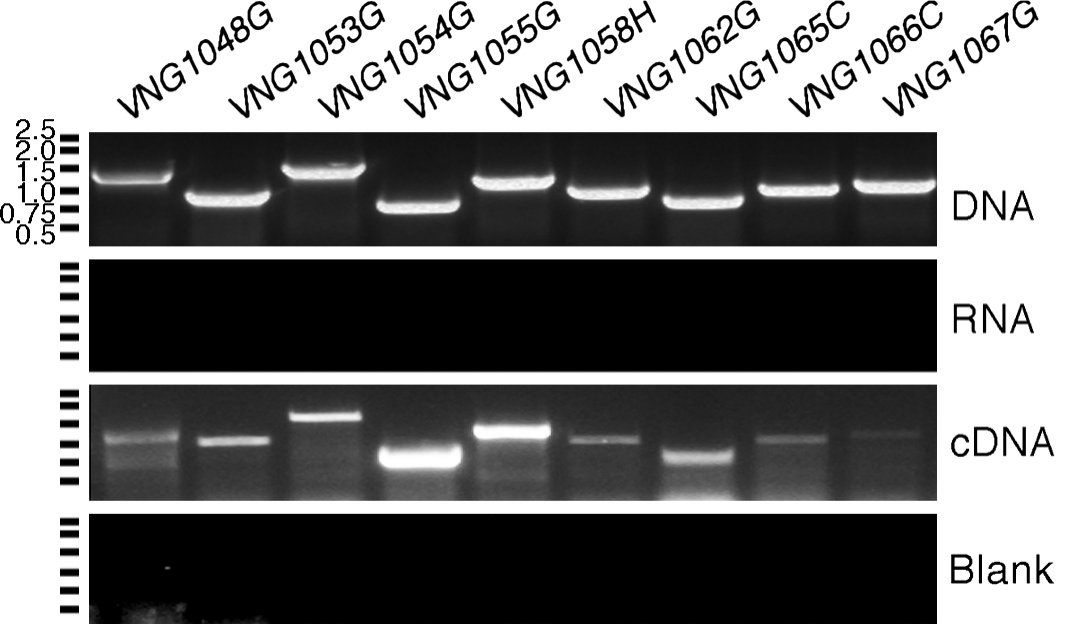
*Halobacterium salinarum agl* gene homologues are transcribed in the native host. RT-PCR was performed using *Hbt. salinarum* DNA, RNA or cDNA as template, together with primers raised against regions at both ends of the gene of interest (Table1S3). In the gel marked blank, no nucleic acid template was included in the reaction. The positions of markers are denoted on the left of each gel, with the corresponding sizes indicated next to the top gel.

While the various *Hbt. salinarum* homologues of *agl* genes can complement *Hfx. volcanii* cells lacking such genes, it remains to be shown that the *Hbt. salinarum* sequences serve similar functions in the native host. Since transcription of a given sequence offers strong support for that open reading frame corresponding to a true gene, RT-PCR was performed for each *Hbt. salinarum* sequence of interest as a first step toward demonstrating their involvement in N-glycosylation in this species. PCR products were obtained for all of the sequences considered within the *VNG1048G*-*VNG1068G* gene cluster when cDNA prepared from RNA extracted from *Hbt. salinarum* cells in exponential phase served as template (Fig4). No products were obtained when DNA or RNA served as template or when no nucleic acids were included in the reaction. On the other hand, no PCR product was obtained for *VNG0318G* using cDNA prepared from cells grown to either exponential or stationary phase.

### In vitro confirmation of VNG1048G and VNG1055G function

**Figure 5:**
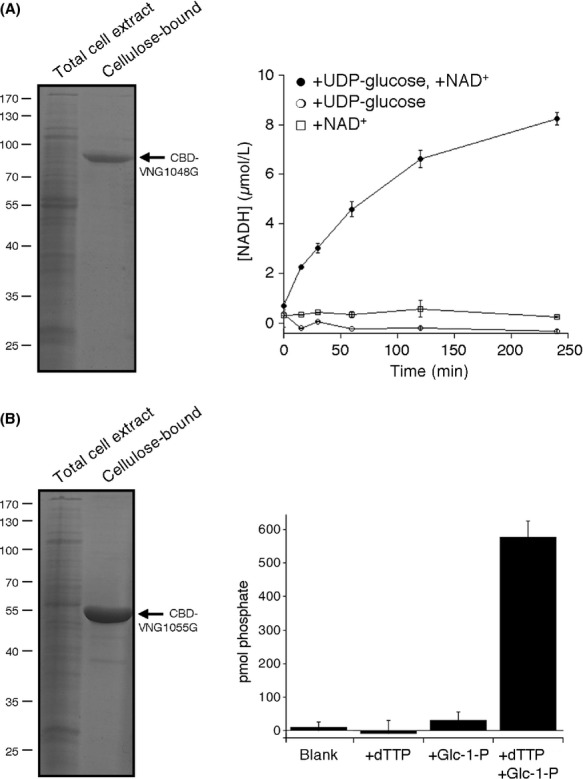
Demonstration of the in vitro activities of VNG1048G and VNG1055G. (A) Left panel: A total lysate of *Haloferax volcanii* transformed to express CBD-tagged VNG-1048G (left lane) and cellulose-purified lysate proteins (right lane) were separated by SDS-PAGE and Coomassie stained. The position of CBD-VNG1048G is indicated, as are the positions of molecular weight markers. Right panel: The activity of cellulose-bound VNG1048G, reflected as an increase in NADH concentration, was assessed as described in Experimental Procedures (B) Left panel: A total lysate of *Hfx. volcanii* transformed to express CBD-tagged VNG-1055G (left lane) and cellulose-purified lysate proteins (right lane) were separated by SDS-PAGE and Coomassie stained. The position of CBD-VNG1055G is indicated, as are the positions of molecular weight markers. Right panel: The activity of cellulose-bound VNG1055G, reflected as an increase in phosphate levels in a 10 *μ*L aliquot of a 500 *μ*L reaction volume following a 60 min incubation, was assessed as described in Experimental Procedures.

To further demonstrate that *Hfx. volcanii* Agl proteins and their *Hbt. salinarum* homologues serve the same roles, selected *Hbt. salinarum* proteins were purified and functionally characterized. Protein selection was based on the availability of biochemical assays for the study of the homologous *Hfx. volcanii* proteins. In this manner, it was shown that *Hbt. salinarum* VNG1048G is a UDP-glucose dehydrogenase, like its *Hfx. volcanii* homologue AglM. Relying on an approach used to study AglM activity (Yurist-Doutsch et al. 2010), the ability of cellulose-bound CBD-tagged VNG1048G (Fig5A, left panel) to transform UDP-glucose into UDP-glucuronic acid in a NAD^+^-dependent manner was confirmed (Fig5A, right panel). To determine whether *Hbt. salinarum* VNG1055G is a glucose-1-phosphate uridyltransferase, able to convert glucose-1-phosphate and UTP into UDP-glucose like its *Hfx. volcanii* homologue AglF (Yurist-Doutsch et al. 2008, 2010), such activity of cellulose-bound CBD-tagged VNG1055G (Fig5B, left panel) was assessed by spectrophotometrically measuring phosphate release. In this manner, it was demonstrated that VNG1055G is a glucose-1-phosphate thymidyltransferase, converting glucose-1-phosphate and dTTP into dTDP-glucose (Fig5B, right panel). In contrast, UTP served as a poor substrate for generating nucleotide-activated glucose in vitro (not shown).

## Discussion

Genome analysis points to N-glycosylation as being a common posttranslational modification in Archaea, with available structural information revealing enormous diversity in terms of glycan composition and architecture. At the same time, largely due to the lack of appropriate molecular tools or difficulties related to culturing in the laboratory, only little is known of the biosynthesis of N-linked glycans in Archaea. With the aim of bridging this gap, the present study addressed the *Hbt. salinarum* pathway responsible for the assembly of one the two N-linked glycans decorating proteins in this species. To do so, the similarity of this *Hbt. salinarum* glycan to a counterpart in *Hfx. volcanii* for which a biosynthetic pathway has been delineated was exploited. Based on this structural resemblance, as well as bioinformatics, genetics, mass spectrometry, and biochemical approaches, this report presents the first outlining of an N-glycosylation pathway in *Hbt. salinarum*, the first noneukaryal organism in which this posttranslational modification was observed (Mescher and Strominger 1976).

**Figure 6:**
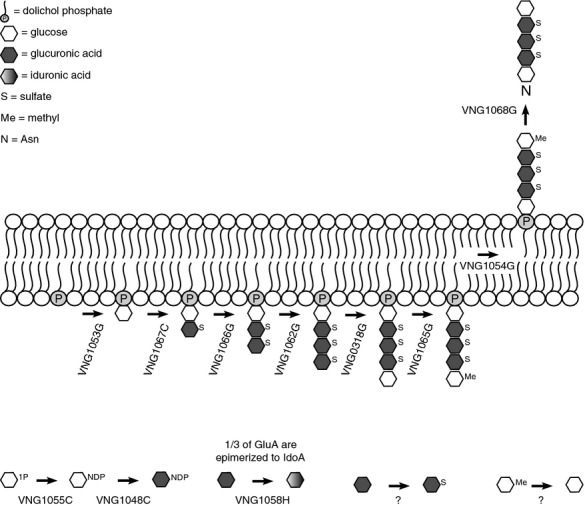
Schematic depiction of the proposed *Halobacterium salinarum* N-glycosylation pathway. See text for details.

In the proposed *Hbt. salinarum* N-glycosylation pathway (Fig6), the findings of the present study are combined those of earlier reports obtained during the pre-genomic era (for review, see Lechner and Wieland 1989). In the putative pathway, the first glucose, the next three glucuronic acids and the final glucose are respectively added to dolichol phosphate by the glycosyltransferases VNG1053G, VNG1067C, VNG1066G, VNG1062C, and VNG0318G. The finding that VNG0318G could replace AglD is unexpected. In *Hfx. volcanii*, AglD adds a nucleotide-activated mannose to dolichol phosphate that is subsequently transferred to the protein-bound tetrasaccharide. In contrast, the final sugar of the glycan N-linked to *Hbt. salinarum* glycoproteins is apparently glucose (Lechner et al. 1985b). Still, the observation that *aglD* and *VNG0318H* are found in highly similar gene clusters would argue the two proteins serve the same role. The reason why VNG1053G, VNG1067C, VNG1066G, and VNG1062C could all replace AglJ is also not clear at this point. It should, however, be noted that the relative efficiencies of each of these replacements was not considered in this study.

In addition to the glycosyltransferases, roles are assigned to the other *Hbt. salinarum* homologues of *Hfx. volcanii* Agl proteins. VNG1065G is predicted to be the methyltransferase responsible for methylating the glucose found at the nonreducing end (Lechner et al. 1985b), while VNG1058H is thought to be the epimerase responsible for converting a third of the glucuronic acids to iduronic acid (Wieland et al. 1986). It should be noted that while evidence for methylation of the final glucose of the pentasaccharide when part of the lipid-linked glycan in *Hbt. salinarum* has been presented (Lechner et al. 1985b), it remains unclear whether iduronic acid is generated as a nucleotide-activated species or rather by epimerization of a glucuronic acid already incorporated into the lipid-linked glycan. Moreover, the precise position(s) of iduronic acid within the glycan is not known. The model further proposes that VNG1055C and VNG1048C act as a glucose-1-phosphate nucleotidyltransferase and a NDP-glucose dehydrogenase, respectively, likely cooperating to convert glucose-1-phosphate into nucleotide-activated glucuronic acid. At the same time, VNG1055C could generate the nucleotide-activated glucose added at the reducing and nonreducing ends of the glycan. While the enzyme responsible for sulfation of the hexuronic acids has yet to be identified, VNG1281H, a hypothetical protein showing over 30% to a sulfotransferase in *Drosophila*, is a possible candidate. Finally, an unknown enzyme is responsible for removing the methyl group attached to the final glucose residue apparently after the lipid-linked glycan has been translocated across the membrane (Lechner et al. 1985). Such translocation is predicted to involve VNG1054G (a homologue of AglR, assigned such a role in *Hfx. volcanii* glycosylation (Kaminski et al. 2012)), while the oligosaccharyltransferase VNG1068G delivers the glycan to target protein Asn residues (Cohen-Rosenzweig et al. 2014).

In addition to similarities in sequence and organization of those genes assigned N-glycosylation roles, *Hfx. volcanii* and *Hbt. salinarum* share other aspects of N-glycosylation. Most striking is the fact that these two organisms represent the only two known examples in which a single protein, the S-layer glycoprotein, is simultaneously modified by two chemically distinct N-linked glycans (Lechner and Wieland 1989; Guan et al. 2012). Nonetheless, differences between that *Hfx. volcanii* N-glycosylation pathway and its predicted *Hbt. salinarum* counterpart described here are apparent. One difference concerns the assembly of the N-linked glycan at the dolichol phosphate level. In *Hfx. volcanii*, the first four pentasaccharide sugars are added to a common dolichol phosphate carrier, while the final pentasaccharide sugar, mannose, is added to a distinct dolichol phosphate (Guan et al. 2010). In *Hbt. salinarum*, the complete pentasaccharide is reportedly found on a single dolichol phosphate carrier (Lechner et al. 1985b). A second difference concerns methylation of the glycan at the lipid-linked stage. In *Hfx. volcanii*, methylation of the hexuronic acid found at the fourth position of N-linked glycan is detected at both the lipid-linked precursor and at the target protein levels. Failure to methylate the dolichol phosphate-bound glycan did not prevent N-glycosylation with a methyl group-lacking tetrasaccharide, although the complete pentasaccharide was not detected (Magidovich et al. 2010). By contrast, in *Hbt. salinarum*, the inability to methylate the final glucose of the dolichol phosphate-linked pentasaccharide prevented N-glycosylation (Lechner et al. 1985b).

In the present study, a series of experiments based on bioinformatics, genetic, and biochemical tools were used to outline a pathway for N-glycosylation in *Hbt. salinarum*. With the availability of a system for deleting genes in this species (Peck et al. 2000), it should be possible to test these predictions.

## Supporting Information

**Figure S1.** *Halobacterium salinarum* VNG1065C can functionally replace *Haloferax volcanii* AglP but is poorly expressed in Δ*aglP* cells. (A) In Δ*aglP* cells transformed to express VNG1065C, an Asn-13-containing S-layer glycoprotein-derived peptide is modified by the first four sugars of the N-linked pentasaccharide normally added to this position. The open circle corresponds to hexose while the full circles correspond to hexuronic acid. The position of the monoisotopic [M+2H]^2+^ ion peak at *m/z* 1143.43 is indicated. (B) LC-ESI MS analysis reveals that the majority of peptide-bound tetrasaccharide is not methylated ([M+2H]^2+^ ion peak at *m/z* 1136.43). (C) MS/MS analysis of the *m/z* 1136.43 species reveals the presence of fragments corresponding to the Asn-13-containing S-layer glycoprotein-derived peptide modified by the first, the first two and the first three pentasaccharide sugars. Hexoses are represented by open circles and hexuronic acids are represented by full circles. (D) A 10 mL aliquot of Δ*aglP* cells transformed to express VNG1065C were lysed and the soluble fraction were incubated with cellulose beads. Aliquots of the total extract and the cellulose-bound proteins were separated by SDS-PAGE and visualized by Coomassie staining (CBB; left panel) or transferred to nitrocellulose and probed with anti-CBD antibodies (α-CBD; right panel). In each panel, the positions of molecular weight markers and the expected position of CBD-VNG1065C are indicated. **Figure S2.** *Haloferax volcanii aglD* and *Halobacterium salinarum VNG0318G* are found in almost identical gene clusters. Gene clusters spanning *Hfx. volcanii HVO_0780*- *HVO_0812* and *Hbt. salinarum VNG0298H*-*VNG330G* were compared. Homologous sequences are connected by vertical lines, with the numbers indicating the percentage in identity at the amino acid level. *Hfx. volcanii aglD* and *Hbt. salinarum VNG0318G* are shaded black. **Figure S3.** *Halobacterium salinarum* VNG0318G can functionally replace *Haloferax volcanii* AglD. LC-ESI MS analysis of an Asn-13-containing S-layer glycoprotein-derived peptide from *Hfx. volcanii* Δ*aglD* cells transformed to express VNG0381G reveals a [M+2H]^2+^ ion peak at *m/z* 1224.47 corresponding to the pentasaccharide-modified peptide. The inset shows the MS/MS profile of the *m/z* 1224.47 species, revealing the presence of the same peptide modified by the mono-, di-, tri-, and tetrasaccharide precursors of the Asn-13-linked pentsaccharide. Hexoses are represented by open circles, hexuronic acids are represented by full circles and mannoses are represented by open circles. **Table S1.** Functional descriptions of *Haloferax volcanii* Agl proteins and their predicted *Halobacterium salinarum* homologues. **Table S2.** BLAST searches of the *Haloferax volcanii* genome using select *Halobacterium salinarum* sequences as queries. **Table S3.** Primers used in this study.

## References

[b1] Abu-Qarn M, Eichler J (2006). Protein N-glycosylation in Archaea: defining *Haloferax volcanii* genes involved in S-layer glycoprotein glycosylation. Mol. Microbiol.

[b2] Abu-Qarn M, Yurist-Doutsch S, Giordano A, Trauner A, Morris HR, Hitchen P (2007). *Haloferax volcanii* AglB and AglD are involved in N-glycosylation of the S-layer glycoprotein and proper assembly of the surface layer. J. Mol. Biol.

[b3] Abu-Qarn M, Eichler J, Sharon N (2008). Not just for Eukarya anymore: N-glycosylation in Bacteria and Archaea. Curr. Opin. Struct. Biol.

[b4] Aebi M (2013). N-linked protein glycosylation in the ER. Biochim. Biophys. Acta.

[b5] Arbiv A, Yurist-Doutsch S, Guan Z, Eichler J (2013). AglQ is a novel component of the *Haloferax volcanii* N-glycosylation pathway. PLoS ONE.

[b6] Cohen-Rosenzweig C, Yurist-Doutsch S, Eichler J (2012). AglS, a novel component of the *Haloferax volcanii* N-glycosylation pathway, is a dolichol phosphate-mannose mannosyltransferase. J. Bacteriol.

[b7] Cohen-Rosenzweig C, Guan Z, Shaanan B, Eichler J (2014). Substrate promiscuity: AglB, the archaeal oligosaccharyltransferase, can process a variety of lipid-linked glycans. Appl. Environ. Microbiol.

[b8] Eichler J (2013). Extreme sweetness: Protein glycosylation in Archaea. Nat. Rev. Microbiol.

[b9] Guan Z, Naparstek S, Kaminski L, Konrad Z, Eichler J (2010). Distinct glycan-charged phosphodolichol carriers are required for the assembly of the pentasaccharide N-linked to the *Haloferax volcanii* S-layer glycoprotein. Mol. Microbiol.

[b10] Guan Z, Naparstek S, Calo D, Eichler J (2012). Protein glycosylation as an adaptive response in Archaea: growth at different salt concentrations leads to alterations in *Haloferax volcanii* S-layer glycoprotein N-glycosylation. Environ. Microbiol.

[b11] Irihimovitch V, Ring G, Elkayam T, Konrad Z, Eichler J (2003). Isolation of fusion proteins containing SecY and SecE, components of the protein translocation complex from the halophilic archaeon *Haloferax volcanii*. Extremophiles.

[b12] Jarrell KF, Ding Y, Meyer BH, Albers SV, Kaminski L, Eichler J (2014). N-Linked glycosylation in Archaea: a structural, functional, and genetic analysis. Microbiol. Mol. Biol. Rev.

[b13] Kaminski L, Abu-Qarn M, Guan Z, Naparstek S, Ventura VV, Raetz CRH (2010). AglJ adds the first sugar of the N-linked pentasaccharide decorating the *Haloferax volcanii* S-layer glycoprotein. J. Bacteriol.

[b14] Kaminski L, Guan Z, Abu-Qarn M, Konrad Z, Eichler J (2012). AglR is required for addition of the final mannose residue of the N-linked glycan decorating the *Haloferax volcanii* S-layer glycoprotein. Biochim. Biophys. Acta.

[b15] Kaminski L, Lurie-Weinberger MN, Allers T, Gophna U, Eichler J (2013). Phylogenetic- and genome-derived insight into the evolution of N-glycosylation in Archaea. Mol. Phylogenet. Evol.

[b16] Lanzetta PA, Alvarez LJ, Reinach PS, Candia OA (1979). An improved assay for nanomole amounts of inorganic phosphate. Anal. Biochem.

[b17] Larkin A, Imperiali B (2011). The expanding horizons of asparagine-linked glycosylation. Biochemistry.

[b18] Lechner J, Wieland F (1989). Structure and biosynthesis of prokaryotic glycoproteins. Annu. Rev. Biochem.

[b19] Lechner J, Wieland F, Sumper M (1985a). Biosynthesis of sulfated saccharides N- glycosidically linked to the protein via glucose. Purification and identification of sulfated dolichyl monophosphoryl tetrasaccharides from halobacteria. J. Biol. Chem.

[b20] Lechner J, Wieland F, Sumper M (1985b). Transient methylation of dolichyl oligosaccharides is an obligatory step in halobacterial sulfated glycoprotein biosynthesis. J. Biol. Chem.

[b21] Magidovich H, Eichler J (2009). Glycosyltransferases and oligosaccharyltransferases in Archaea: putative components of the N-glycosylation pathway in the third domain of life. FEMS Microbiol. Lett.

[b22] Magidovich H, Yurist-Doutsch S, Konrad Z, Ventura VV, Hitchen PG, Dell A (2010). AglP is a S-adenosyl-L-methionine-dependent methyltransferase that participates in the N-glycosylation pathway of *Haloferax volcanii*. Mol. Microbiol.

[b23] Mescher MF, Strominger JL (1976). Purification and characterization of a prokaryotic glucoprotein from the cell envelope of *Halobacterium salinarium*. J. Biol. Chem.

[b24] Morag E, Lapidot A, Govorko D, Lamed R, Wilchek M, Bayer EA (1995). Expression, purification, and characterization of the cellulose-binding domain of the scaffoldin subunit from the cellulosome of *Clostridium thermocellum*. Appl. Environ. Microbiol.

[b25] Nothaft H, Szymanski CM (2013). Bacterial protein N-glycosylation: new perspectives and applications. J. Biol. Chem.

[b26] Peck RF, DasSarma S, Krebs MP (2000). Homologous gene knockout in the archaeon *Halobacterium salinarum* with ura3 as a counterselectable marker. Mol. Microbiol.

[b27] Plavner N, Eichler J (2008). Defining the topology of the N-glycosylation pathway in the halophilic archaeon *Haloferax volcanii*. J. Bacteriol.

[b28] Wieland F (1988). Structure and biosynthesis of prokaryotic glycoproteins. Biochimie.

[b29] Wieland F, Paul G, Sumper M (1985). Halobacterial flagellins are sulfated glycoproteins. J. Biol. Chem.

[b30] Wieland F, Lechner J, Sumper M (1986). Iduronic acid: constituent of sulphated dolichyl phosphate oligosaccharides in halobacteria. FEBS Lett.

[b31] Yurist-Doutsch S, Eichler J (2009). Manual annotation, transcriptional analysis and protein expression studies reveal novel genes in the *agl* cluster responsible for N-glycosylation in the halophilic archaeon *Haloferax volcanii*. J. Bacteriol.

[b32] Yurist-Doutsch S, Abu-Qarn M, Battaglia F, Morris HR, Hitchen PG, Dell A (2008). *aglF*
*aglG* and *aglI*, novel members of a gene cluster involved in the N-glycosylation of the *Haloferax volcanii* S-layer glycoprotein. Mol. Microbiol.

[b33] Yurist-Doutsch S, Magidovich H, Ventura VV, Hitchen PG, Dell A, Eichler J (2010). N-glycosylation in Archaea: on the coordinated actions of *Haloferax volcanii* AglF and AglM. Mol. Microbiol.

