# Supplementary material for: Deciphering a pathway of Halobacterium salinarum N-glycosylation
Source: Microbiologyopen. 2014 Dec 2;4(1):28–40. doi: 10.1002/mbo3.215 (PMC4335974; doi:10.1002/mbo3.215)
Supplement: Supplementary file 1 [file mbo30004-0028-sd1.pdf]

Table S1 – Functional descriptions of *Hfx. volcanii* Agl proteins and their predicted *Hbt. salinarum* homologues

Hfx. volcanii		***Hbt. salinarum***	
**Protein**	**Demonstrated function**	**Protein**	**Currently annotated function**
AglJ	glycosyltransferase	VNG1053G	glycosyltransferase
AglP	methyltransferase	VNG1065C	methyltransferase
AglQ	isomerase	VNG1058H	hypothetical protein
AglE	glycosyltransferase	VNG1062G	rhamnosyltransferase
AglR	flippase/flippase-related	VNG1054G	probable transport protein
AglF	glucose-1-phosphate uridylyltransferase	VNG1055G	glucose-1-phosphate thymidylyltransferase
AglI	glycosyltransferase	VNG1066C	glycosyltransferase
AglG	glycosyltransferase	VNG1067G	glycosyltransferase
AglB	oligosaccharyltransferase	VNG1068G	oligosaccharyltransferase
AglM	UDP-glucose dehydrogenase	VNG1048G	UDP-glucose dehydrogenase
AglD	glycosyltransferase	VNG0318G	glycosyltranferase

Table S2 – BLAST searches of the *Hfx. volcanii* genome using select *Hbt. salinarum* sequences as queries

***Hbt. salinarum* query**	***Hfx. volcanii* hit**	**E-value**	**Score**	**% coverage**
VNG1053G	AglJ	6e-124	363	98
VNG1065C	AglP	4e-29	115	83
VNG1058H	AglQ	2e-66	221	76
VNG1062G	AglE	4e-40	141	71
VNG1054G	AglR	1e-41	159	98
VNG1055G	AglF	2e-142	405	96
VNG1066C	AglI	4e-98	297	94
VNG1067G	AglG	8e-155	444	100
VNG1068G	AglB	0	860	97
VNG1048G	AglM	2e-163	474	94

Table S3 – Primers used in this study

Primer	Description	Sequence
VNG1048G NdeI for	Forward primer introducing an NdeI site at the start of *VNG1048G*	ccccatatgGACGTGAGCATCGTTGGGAGTGGG
VNG1048G StuI rev	Reverse primer introducing a StuI site at the end of *VNG1048G*	gggaggcctCTACCAGGTGAGCCCGTCGTAGGTC
VNG1053G BglII for	Forward primer introducing a BglII site at the start of *VNG1053G*	cccagatctATGAGCGAGGAGTACGAG
VNG1053G KpnI rev	Reverse primer introducing a KpnI site at the end of *VNG1053G*	cccggtaccTCACTTGTTCAGGCGC
VNG1054G NdeI for	Forward primer introducing an NdeI site at the start of *VNG1054G*	ccccatatgAGATACGGTCGAACGTCG
VNG1054G StuI rev	Reverse primer introducing a StuI site at the end of *VNG1054G*	gggaggcctTTATCCGTCCCATCGGTGG
VNG1055G NdeI for	Forward primer introducing an NdeI site at the start of *VNG1055G*	ccccatatgCAAGCAGTCGTGCTCGCGG
VNG1055G StuI rev	Reverse primer introducing a StuI site at the end of *VNG1055G*	gggaggcctTCACTCGTTGCTCACTGCGTCG
VNG1058H NdeI for	Forward primer introducing an NdeI site at the start of *VNG1058H*	cccCATATGCCGTCTAGTTCGAACCGCCATCTCACC
VNG1058H StuI rev	Reverse primer introducing a StuI site at the end of *VNG1058H*	gggAGGCCTTCAGTGTAGGTCTACGTTC
VNG1062G BglII for	Forward primer introducing a BglII site at the start of *VNG1062G*	cccagatctATGCCTGATTCCCCGTTC
VNG1062G KpnI rev	Reverse primer introducing a KpnI site at the end of *VNG1062G*	cccggtaccTCAGGTCGTGTCTCCG
VNG1065C NdeI for	Forward primer introducing an NdeI site at the start of *VNG1065C*	ccccatatgGCTGAATCGTTACTTGCCTCGGTTTCG
VNG1065C StuI rev	Reverse primer introducing a StuI site at the end of *VNG1065C*	gggaggcctTCATCGGTCATCTACTGTAGCAAGTAC
VNG1066C BglII for	Forward primer introducing a BglII site at the start of *VNG1066C*	cccagatctATGACCGATGAATCCGAC
VNG1066C KpnI rev	Reverse primer introducing a KpnI site at the end of *VNG1066C*	cccggtaccCTAACTCGTAGGCGC
VNG1067G BglII for	Forward primer introducing a BglII site at the start of *VNG1067G*	cccagatctATGCCCCAAAGCACAATC
VNG1067G KpnI rev	Reverse primer introducing a KpnI site at the end of *VNG1067G*	cccggtaccCTACGAGTTAGCGTGC
VNG0318G NdeI for	Forward primer introducing a BglII site at the start of *VNG0318G*	gggcatatgAGCGAGACGGCCGTCGAGGTGAG
VNG0318G KpnI rev	Reverse primer introducing a KpnI site at the end of *VNG0318G*	cccggtaccTCAGTCGTCGACGCCCACC

Genomic sequences are in capitals.

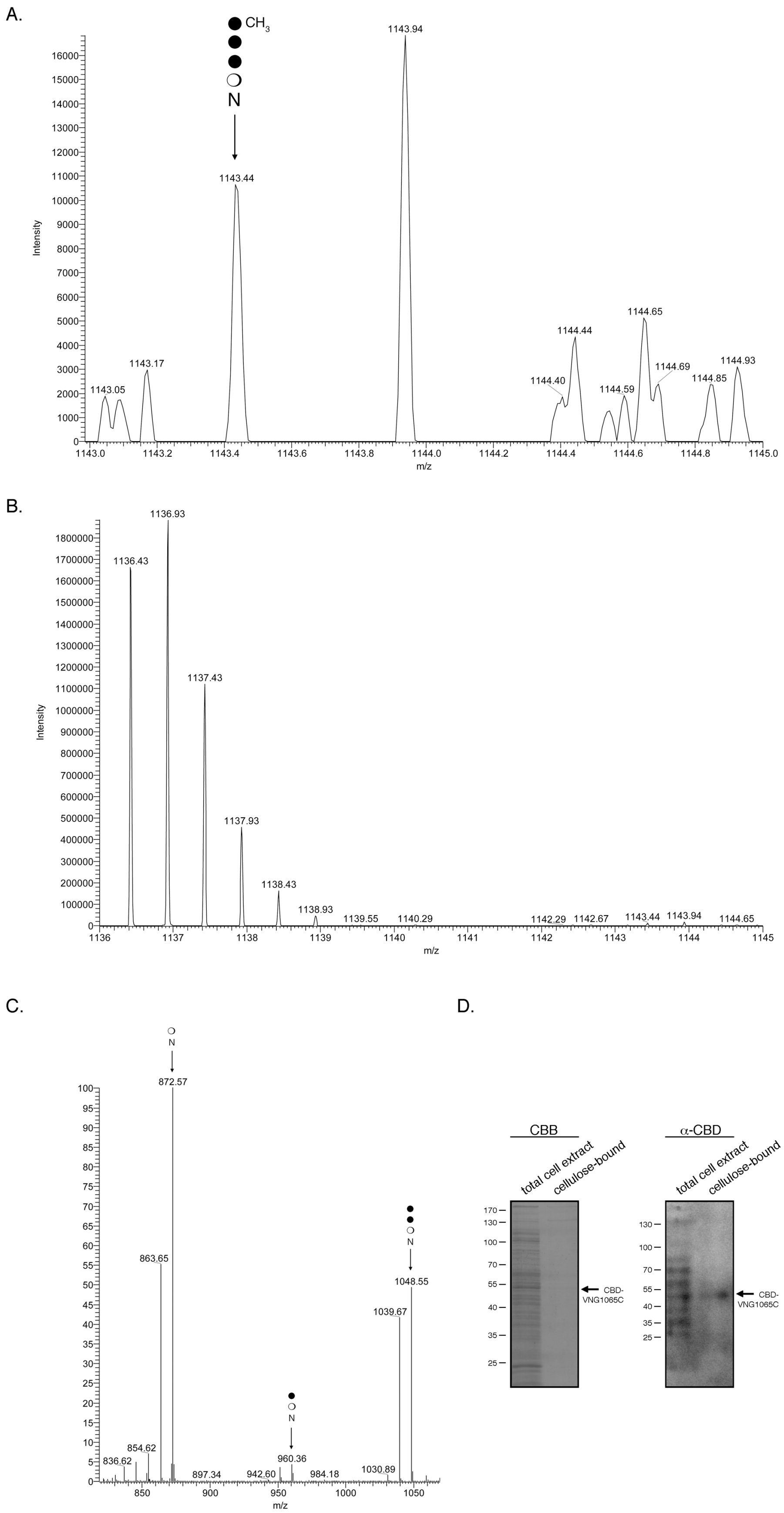

A.
CH3
N
1143.44
1143.94
1143.05
1143.17
1144.40
1144.44
1144.59
1144.65
1144.69
1144.85
1144.93
Intensity
m/z
B.
1136.43
1136.93
1137.43
1137.93
1138.43
1138.93
1139.55
1140.29
1142.29
1142.67
1143.44
1143.94
1144.65
Intensity
m/z
C.
N
872.57
863.65
854.62
836.62
897.34
942.60
960.36
984.18
1030.89
1039.67
1048.55
m/z
D.
CBB
α-CBD
total cell extract
cellulose-bound
CBD-VNG1065C

Fig S1 – *Hbt. salinarum* VNG1065C can functionally replace *Hfx. volcanii* AglP but is poorly expressed in *ΔaglP* cells. A. In *ΔaglP* cells transformed to express VNG1065C, an Asn-13-containing S-layer glycoprotein-derived peptide is modified by the first four sugars of the N-linked pentasaccharide normally added to this position. The open circle corresponds to hexose while the full circles correspond to hexuronic acid. The position of the monoisotopic $[M+2H]^{2+}$ ion peak at (*m/z* 1143.43) is indicated. B. LC-ESI MS analysis reveals that the majority of peptide-bound tetrasaccharide is not methylated ($[M+2H]^{2+}$ ion peak at *m/z* 1136.43). C. MS/MS analysis of the *m/z* 1136.43 reveals the presence of fragments corresponding to the Asn-13-containing S-layer glycoprotein-derived peptide modified by the first, the first two and the first three pentasaccharide sugars. Hexoses are represented by open circles and hexuronic acids are represented by full circles. D. A 10 ml aliquot of *ΔaglP* cells transformed to express VNG1065C were lysed and the soluble fraction were incubated with cellulose beads. Aliquots of the total extract and the cellulose-bound proteins were separated by SDS-PAGE and visualized by Coomassie staining (CBB; left panel) or transferred to nitrocellulose and probed with anti-CBD antibodies (α-CBD; right panel). In each panel, the positions of molecular weight markers and the expected position of CBD-VNG1065C are indicated.

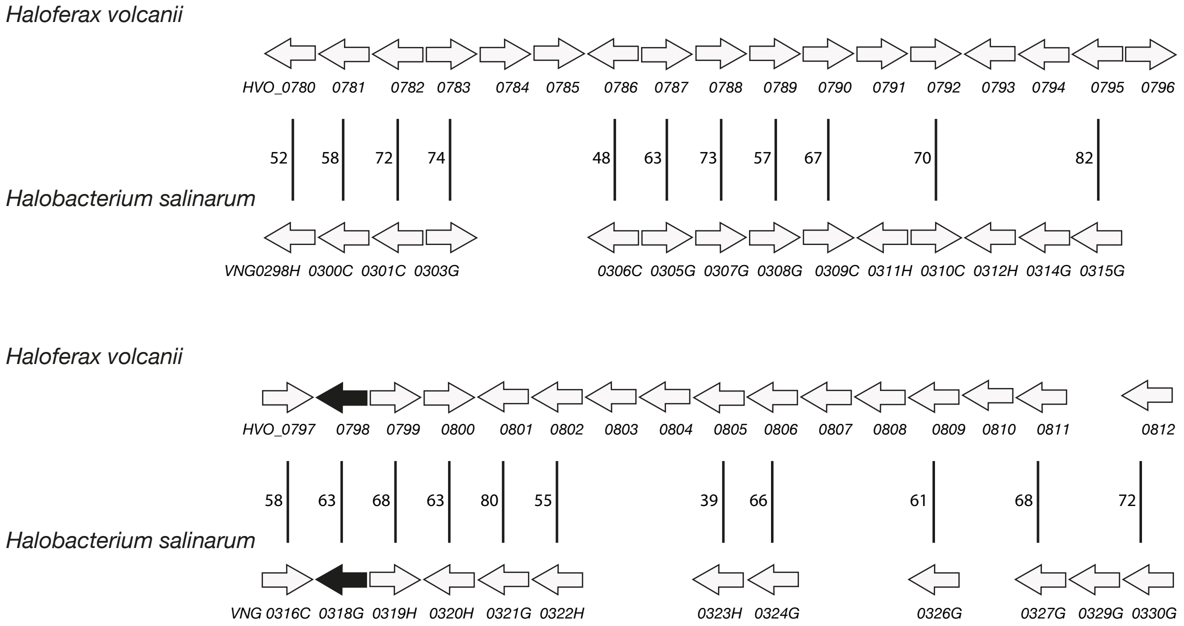

Fig S2 – *Hfx. volcanii aglD* and *Hbt. salinarum VNG0318G* are found in almost identical gene clusters. Gene clusters spanning *Hfx. volcanii HVO_0780- HVO_0812* and *Hbt. salinarum VNG0298H-VNG330G* were compared. Homologous sequences are connected by vertical lines, with the numbers indicating the percentage in identity at the amino acid level. *Hfx. volcanii aglD* and *Hbt. salinarum VNG0318G* are shaded black.

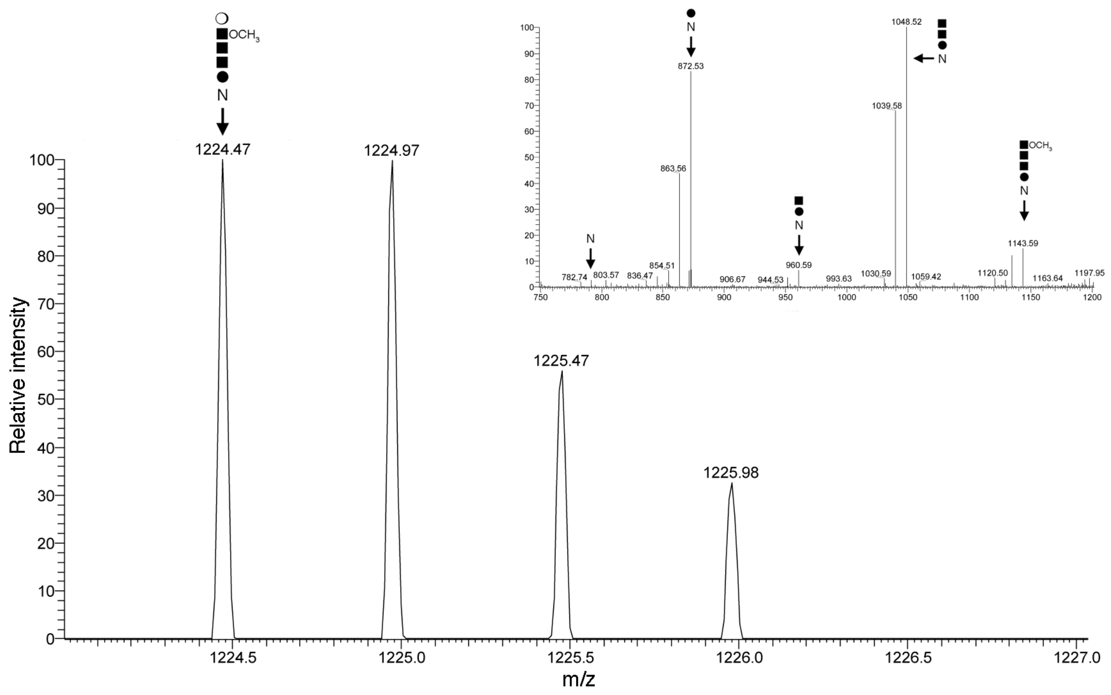

Fig S3 - *Hbt. salinarum* VNG0318G can functionally replace *Hfx. volcanii* AglD. LC-ESI MS analysis of an Asn-13-containing S-layer glycoprotein-derived peptide from *Hfx. volcanii ΔaglD* cells transformed to express VNG0381G reveals a $[M+2H]^{2+}$ ion peak at *m/z* 1224.47 corresponding to the pentasaccharide-modified peptide. The inset shows the MS/MS profile of the *m/z* 1224.47 species, revealing the presence of the same peptide modified by the mono-, di-, tri- and tetrasaccharide precursors of the Asn-13-linked pentsaccharide. Hexoses are represented by open circles, hexuronic acids are represented by full circles and mannoses are represented by open circles.
